# The Organelle Genomes of *Hassawi* Rice (*Oryza sativa* L.) and Its Hybrid in Saudi Arabia: Genome Variation, Rearrangement, and Origins

**DOI:** 10.1371/journal.pone.0042041

**Published:** 2012-07-31

**Authors:** Tongwu Zhang, Songnian Hu, Guangyu Zhang, Linlin Pan, Xiaowei Zhang, Ibrahim S. Al-Mssallem, Jun Yu

**Affiliations:** 1 Joint Center for Genomics Research (JCGR), King Abdulaziz City for Science and Technology and Chinese Academy of Sciences, Riyadh, Kingdom of Saudi Arabia and Beijing, China; 2 CAS Key Laboratory of Genome Sciences and Information, Beijing Institute of Genomics, Chinese Academy of Sciences, Beijing, China; 3 James D. Watson Institute of Genome Sciences, College of Life Science, Zhejiang University, Hangzhou, China; 4 Department of Biotechnology, College of Agriculture and Food Sciences, King Faisal University, AI-Hssa, Hofuf, Kingdom of Saudi Arabia; University of Arizona, United States of America

## Abstract

*Hassawi* rice (*Oryza sativa* L.) is a landrace adapted to the climate of Saudi Arabia, characterized by its strong resistance to soil salinity and drought. Using high quality sequencing reads extracted from raw data of a whole genome sequencing project, we assembled both chloroplast (cp) and mitochondrial (mt) genomes of the wild-type *Hassawi* rice (*Hassawi-1*) and its dwarf hybrid (*Hassawi-2*). We discovered 16 InDels (insertions and deletions) but no SNP (single nucleotide polymorphism) is present between the two *Hassawi* cp genomes. We identified 48 InDels and 26 SNPs in the two *Hassawi* mt genomes and a new type of sequence variation, termed reverse complementary variation (RCV) in the rice cp genomes. There are two and four RCVs identified in *Hassawi-1* when compared to *93–11* (*indica*) and *Nipponbare* (*japonica*), respectively. Microsatellite sequence analysis showed there are more SSRs in the genic regions of both cp and mt genomes in the *Hassawi* rice than in the other rice varieties. There are also large repeats in the *Hassawi* mt genomes, with the longest length of 96,168 bp and 96,165 bp in *Hassawi-1* and *Hassawi-2*, respectively. We believe that frequent DNA rearrangement in the *Hassawi* mt and cp genomes indicate ongoing dynamic processes to reach genetic stability under strong environmental pressures. Based on sequence variation analysis and the breeding history, we suggest that both *Hassawi-1* and *Hassawi-2* originated from the Indonesian variety *Peta* since genetic diversity between the two *Hassawi* cultivars is very low albeit an unknown historic origin of the wild-type *Hassawi* rice.

## Introduction

Rice (*Oryza sativa* L.) is a grass species of plants, providing staple food for over half of the world populations. Aside from being one of the top three major cereal crops, rice is also widely used as a model plant for genetic studies [1]. The cultivated rice has two main subspecies, *indica* and *japonica*, which are estimated to be separated about 0.05–0.44 mya [2]. The *Hassawi* rice (*Oryza sativa* L.) is a landrace adapted to the climate of Eastern Saudi Arabia. It is characterized by strong adaptability to soil salinity and drought [3], [4]. However, it bears some undesired characteristics such as susceptibility for lodging, delayed maturity, and photoperiod sensitivity. In Saudi Arabia, there are two *Hassawi* cultivars, which provide a staple carbohydrate source [3]. One of the cultivars, *Hassawi-1* is the wild-type originated from an *indica* ancestor and the other cultivar *Hassawi-2* is a hybrid between *Hassawi-1* and IR1112 (according to the breeding record, its maternal parent is IR262-43-8-11, a cultivar originated from *Peta*, an *indica* variety from Indonesia). However, until now, few studies have been carried out on genetics and genomics about this valuable rice variety and its related cultivars, and there has been a limited literature about the genetic background of those *Hassawi* rice. The organelle genome sequences of *Hassawi* rice are very helpful for understanding the inheritance of this cultivar and its future breeding research.

The complete chloroplast (cp) and mitochondrion (mt) genomes of both *indica* and *japonica* are published [2], [5]–[7], and a comparative analysis showed that the gene order and essential gene content are highly conserved for most cp genomes [8]. In contrast, plant mt genomes are known to be more complex than those of chloroplasts. The mt-encoded genes are highly conserved, but their gene order, genomes structure, and genome size are highly variable among plant species [2], [9], [10]. Genetic markers have played a major role in our understanding of heritable traits, serving as landmarks for genes and their variations. With the increasing application of next-generation sequencing technologies, there is a rapid growth in information on genetic polymorphisms [11] albeit minor hindrance of sequencing errors [12]. As the most abundant genetic markers, the discovery of both single nucleotide polymorphism (SNP) and insertion or deletion (InDel) is of paramount importance for marker-assisted crop breeding and genetic studies. Simple sequence repeats (SSRs), also known as microsatellites, are also abundant across plant organellar genomes. SSR-based markers can be developed to be a useful tool in determining the maternal origin of rice varieties and for phylogenetic studies [13]. SNPs, InDels, and SSRs of organellar genomes are all invaluable as genetic markers for plant genetics.

**Table 1:** General features of cp and mt genomes among the four rice cultivars.

		Size (bp)	GC (%)	Gene number (total/protein/tRNA/rRNA)	Coding (%)	Repeat (%)	SSR (%)	Cp-derived^1^ (%)
Chloroplast	*Hassawi-1*	134448	39.0	136/91/37/8	54.9	1.2	4.3	–
	*Hassawi-2*	134459	39.0	136/91/37/8	54.9	1.2	4.3	–
	*93–11*	134496	39.0	136/91/37/8	54.9	1.2	4.4	–
	*Nipponbare*	134525	39.0	136/91/37/8	54.9	1.2	4.4	–
Mitochondrion	*Hassawi-1*	454820	43.9	102/69/27/6	15.9	54.2	3.6	5.9
	*Hassawi-2*	454894	43.8	101/67/28/6	15.6	54.2	3.6	5.9
	*93–11*	491515	43.8	93/54/33/6	12.4	58.5	3.5	6.9
	*Nipponbare*	490520	43.9	84/56/22/6	10.8	59.7	3.6	6.9

Note: 1. Cp-derived stands for sequences that are homologous to those of rice cp genomes.

**Figure 1:**
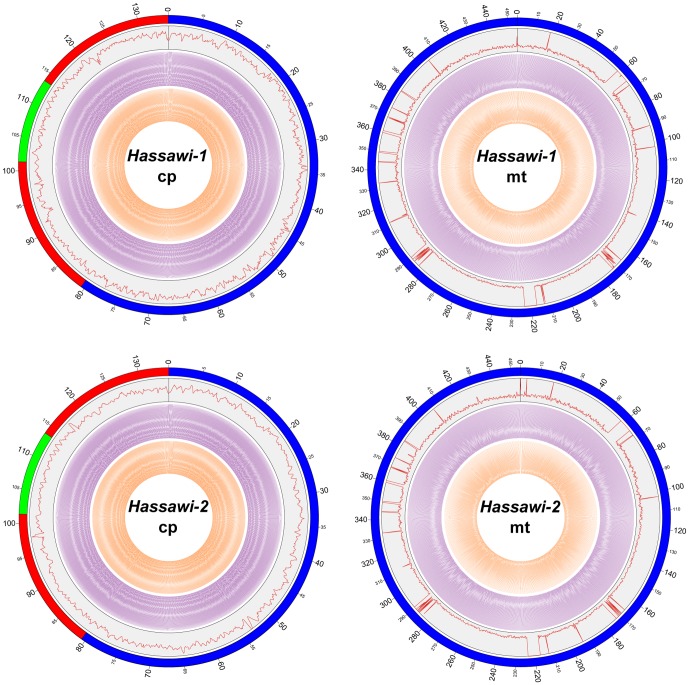
Circular representation of the cp and mt genome assemblies of both *Hassawi-1* and *Hassawi-2*. Circle display (from the outside): (1) physical map scale in kilobase pairs (in cp genome, LSC region in blue, SSC region in green, and IRs regions in red); (2) read depths of the 454 sequencing data in plum (step size: 100 bp in cp genome and 200 bp in mt genome; cp assembly: range 200–1325 in *Hassawi-1* and range 200–2230 in *Hassawi-2*; mt assembly: range 0–500); (3) SOLiD mate-pair read validation with the 0.5–1 kb insert library in purple (insert size 600–800 bp and step size 100 bp in the cp assembly and 450 bp in the mt assembly); (4) SOLiD mate-pair read validation with 1–3 kb library in orange (insert size 1400–1600 bp and step size 150 bp in the cp assembly and 700 bp in the mt assembly). The high variance in read depth of the mt genome results from the regions of cp-derived sequences. This figure is generated by the Circos program.

We recently began to sequence the genomes of two *Hassawi* cultivars using next generation sequencing platforms (both 454 GS FLX and SOLiD 4.0). Using our recently published procedure for plant organellar genome assembly [14], we finished both cp and mt genomes of *Hassawi-1* and *Hassawi-2.* Our in-depth comparative analysis of the organellar genomes revealed the genome variations of SNP, InDel, SSR, reverse complementary variation (RCV) and repeats among *Hassawi-1*, *Hassawi-2*, *indica 93–11* and *japonica Nipponbare.* Based on those sequence variation analysis and the breeding history, we confirmed that both *Hassawi-1* and *Hassawi-2* were originated from the Indonesian variety, which provide important information for future genetic studies of this unique rice variety.

**Figure 2:**
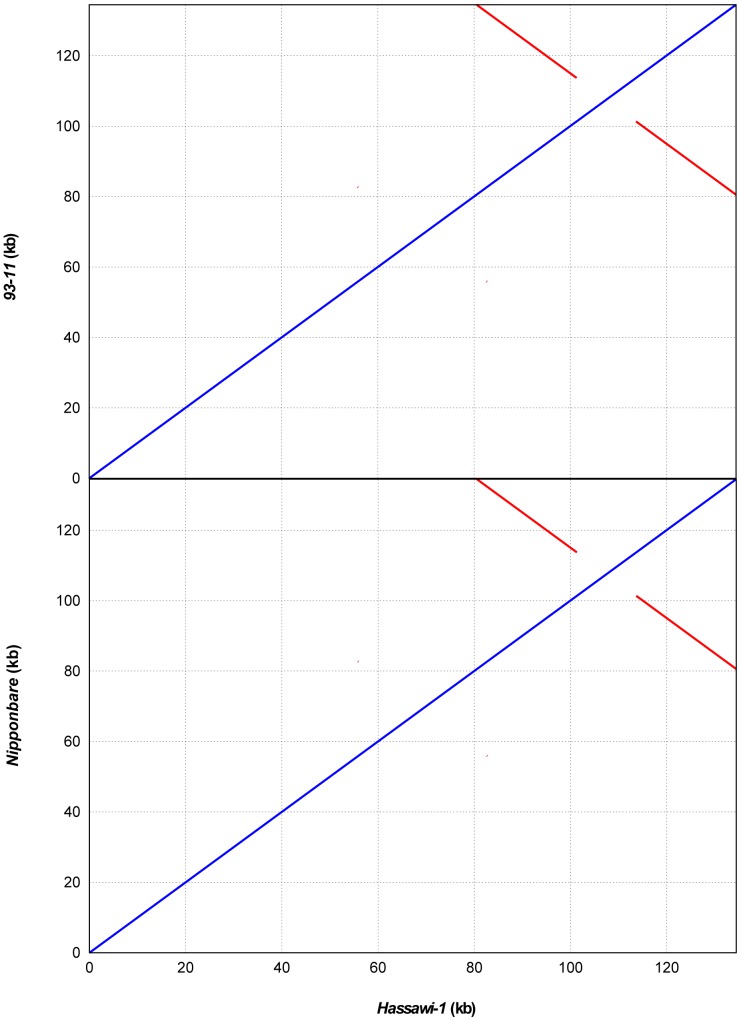
Dot matrix alignment of cp genomes between *Hassawi-1* and *93-11* (top) and between *Hassawi-1* and *Nipponbare* (bottom). The blue and red lines show direct and reverse matches, respectively. The red lines indicate IR regions in cp genomes.

## Results and Discussion

### Genome Assembly Results

The original raw data from the whole genome shotgun sequencing projects contain a large amount of reads from cp and mt genomes, which can be assembled into complete genome sequences independently [2], [15]. We have developed an efficient procedure for plant organellar genome assembly, which based on whole genome data from the 454 sequencing platform [14]. Using this procedure, we have successfully assembled the complete cp and mt genome sequences of *Boea hygrometrica* from the whole genome sequencing data [16]. With the same method, we used this procedure to assemble the cp and mt genomes of both *Hassawi-1* and *Hassawi-2*.

Using 454 sequencing platform, We totally got 7 runs (2.37 Gbp)and 11 runs (3.49 Gbp) for *Hassawi-1* and *Hassawi-2*, respectively (our unpublished data). The raw data quality was good with 95% bases above Q40 and with the peak of the average read quality above 30 for both *Hassawi-1* and *Hassawi-2*. There were totally 419,015 reads (136 Mbp) and 550,696 reads (207 Mbp) filtered as reads belong to cp genome in *Hassawi-1* and *Hassawi-2*, respectively. Using Roche Newbler software, we *de novo* assembled those reads and constructed the contig graphs for whole cp genome. There were 32 and 131 contigs with total length 134,448 bp and 134,459 bp composed complete cp genome of *Hassawi-1* and *Hassawi-2*, respectively (**Figure S1 and S2**). The assembly of mt genome was more complex than cp genome. Using all the sequencing raw data, we assembled 278,498 and 235,389 contigs with average length 1,061 bp and 1,417 bp in *Hassawi-1* and *Hassawi-2*, respectively. With the same method of assembling mt genome of *Boea hygrometrica* [14], [16], we filtered and construed contig graphs belong to mt genome with the reference of mt genomes of other rice (**Table 1**). There were 117 and 213 contigs with total length 454,820 bp and 454,894 bp composed complete mt genome of *Hassawi-1* and *Hassawi-2*, respectively (**Figure S3 and S4**). In order to assess the cp and mt assembly quality for both *Hassawi-1* and *Hassawi-2*, we mapped both the 454 raw data and the SOLiD mate pair data (data unpublished) with different insert size to the assembled cp and mt genomes. The result showed that in all assembly, there were no gap between two connecting contigs and all the contig order were supported by mate pair reads (**Figure 1**).

**Figure 3:**
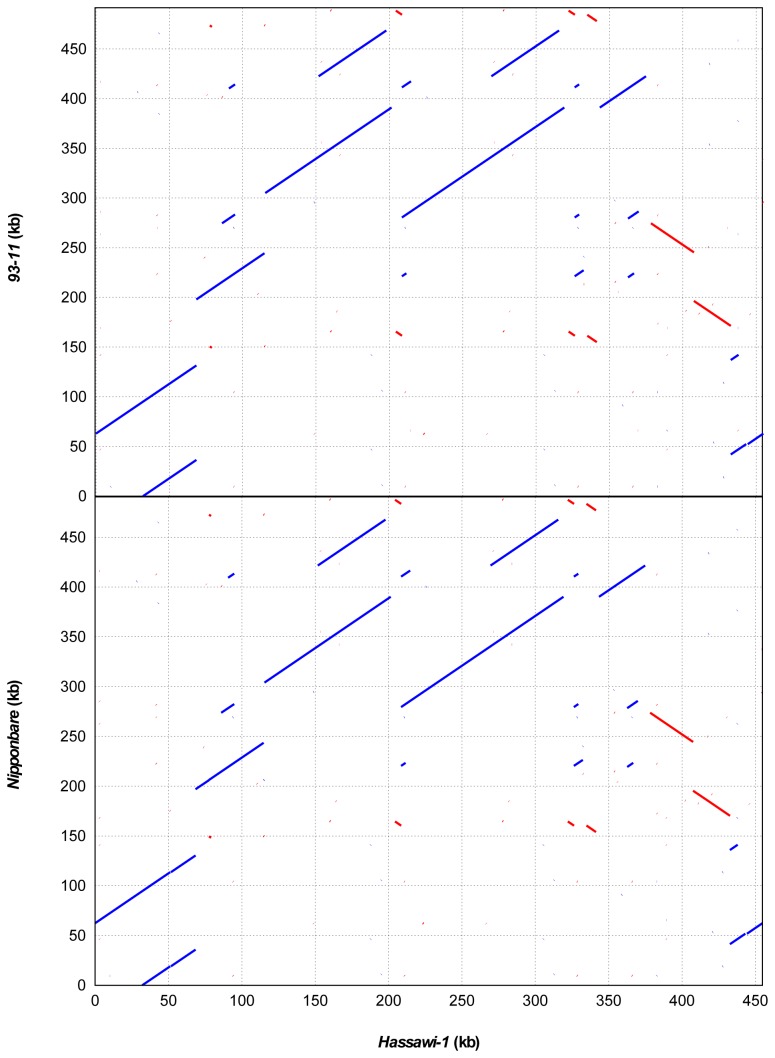
Dot matrix alignment of mt genomes between *Hassawi-1* and *93-11* (top) and between *Hassawi-1* and *Nipponbare* (bottom). The blue and red lines show direct and reverse matches, respectively.

### Genome Features of the Two Hassawi Rice Cultivars

The cp genome is in general composed of a single circular molecule with a quadripartite structure, which includes a large single copy region (LSC) and a small single copy region (SSC), separated by two copies of inverted repeats (IRs) [17], [18]. The *Hassawi-1* cp genome has 134,448 bp in length and a GC content of 39%. 58.8% of its genes are located in LSC region (80,513 bp, covering 59.9% of the cp genome). All rRNA and 16 tRNA genes reside in IR regions (41,590 bp, covering 30.9% of the cp genome). The SSC region (12,345 bp, 9.2%) includes most NADH oxidoreductases. In plant cp genomes, gene content, order, and organization are highly conserved and their inheritance is always maternal, which is different from nuclear genomes [17], [19]. Gene number (136), coding fraction (54.9%), and repeat content (1.2%) of the two *Hassawi* rice are identical (**Table 1**). The sequence alignment of *Hassawi-1* to *93–11* and *Nipponbare* shows excellent colinearity (**Figure 2**). Other than what is in IR regions, we failed to identify any sequence repeats greater than 1 kb in the *Hassawi* cp genomes.

The mt genome is much larger and more complex than the cp genome [20]. Moreover, mt genomes of seed plants are unusually variable in size at least in an order of magnitude, and much of these variations occur within a single family [21]. In this study, we observed that the length of *Hassawi* mt genomes is obviously different from those of *93–11* and *Nipponbare* (**Table 1**). The wild-type *Hassawi* rice has a circular DNA molecule with 454,820 bp in length, which is smaller than both *93–11* (491,515 bp) and *Nipponbare* (490,520 bp). However, compared to *93–11* and *Nipponbare*, the mt genome of *Hassawi-1* has a larger coding region (15.9%) and smaller repeat region (54.2%). Unlike cp genomes, most mt genomes are non-coding or functionally unknown. The functional genes in plant mt genomes are conserved between *Hassawi* and other varieties, such as NADH dehydrogenase and cytochrome c oxidase. The mt genomic structure of *Hassawi-1* is very different from that of *93–11* and *Nipponbare*. Alignment plots among them show multiple recombination events (**Figure 3**). There are some large repeats (>10 kb) in the rice mt genomes, which were also found in other seed plants [22], [23]. The percentage of cp-derived sequences in the *Hassawi* mt genomes is lower than that of other varieties.

### Cp Genome Variations

**Figure 4:**
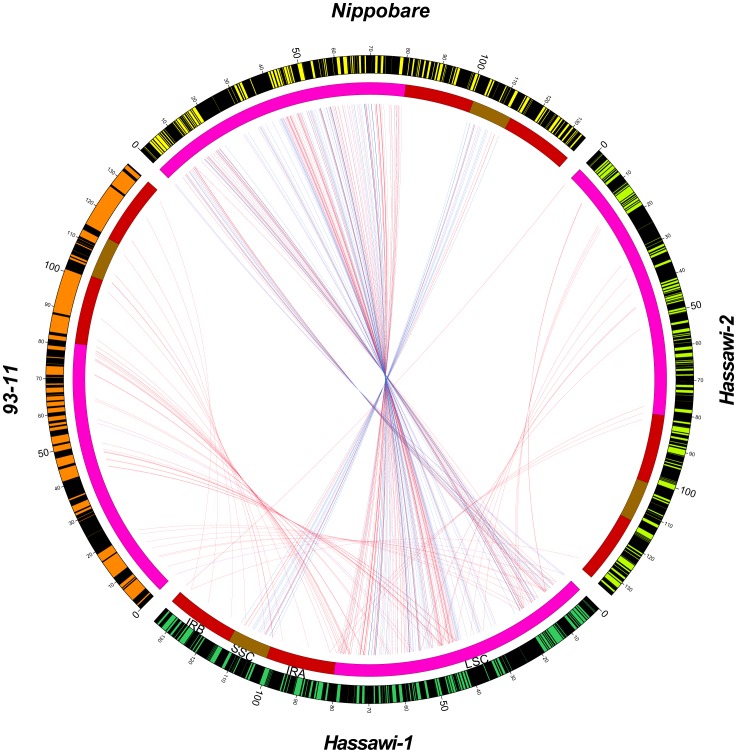
Circos diagram illustrating SNP and InDel distributions in cp genomes of *Hassawi-1* and the other three cultivars. The first circle (from outside) displays genomes (color-coding) and genes (blocks). The second circle displays genomic regions including SSC, LSC, IRA, and IRB. The connecting lines inside the circles show SNPs (blue) and InDels (red) between two genomes.

There are two basic categories of sequence variations with regard to varieties and subspecies when assessing polymorphisms in organellar genomes; one is intraspecific or intravarietal, where variations within a variety or subspecies are identified, and the other is interspecific or intervarietal, where variations between two varieties or subspecies are defined [2], [5]. Comparing cp genomes of *Hassawi-1* and *Hassawi-2*, we detected eleven insertion and five deletion events (**Table 2**), which resulted in an 11-bp difference overall. Among all 16 InDel events, we detected one deletion and two insertions as intravarietal InDels based on an analysis of the sequencing reads. All these InDels are located in intergenic regions. Three InDels (D-2, I-4 and I-4 with positions of 36,491, 80,517, and 134,446 in *Hassawi-1,* respectively) are larger than 1 bp and are all located in LSC region and they are candidate genetics marks for distinguishing wild-type *Hassawi* rice from its hybrid. We did not observe any SNP between *Hassawi-1* and *Hassawi-2* (**Figure 4**).

**Table 2:** InDels of cp genomes between *Hassawi* rice and its hybrid.

InDel^1^	Position in *Hassawi-1*	Position in *Hassawi-2*	Sequence	Intravarietal InDel^2^	Gene location	Region
I-1	8069	8076	A	Y	*trnS-GCU/psbD*	LSC
D-1	8214	8220	T	N	*trnS-GCU/psbD*	
I-1	17031	17038	A	N	*psbM/petN*	
D-1	17734	17740	T	Y	*petN/trnC-GCA*	
I-1	18586	18593	A	Y	*trnC-GCA/rpoB*	
D-1	31973	31979	T	N	*atpI/atpH*	
D-2	36491	36496	TT	N	*trnR-UCU/psaB*	
I-1	41448	41453	A	N	*psaA/ycf3*	
I-1	46123	46129	T	N	*trnT-UGU/trnL-UAA*	
I-1	46432	46439	A	N	*trnT-UGU/trnL-UAA*	
D-1	53261	53267	A	N	*atpB/rbcL*	
I-1	78351	78358	T	N	*rpl16/rps3*	
I-4	80517	80525	TCTT	N	*rpl22/rps19*	
I-1	82608	82620	T	N	*rpl2/rpl23*	IRA
I-1	132357	132370	A	N	*rpl23/rpl2*	IRB
I-4	134446	1	GAAA	N	*rps19/psbA*	LSC

1 *Hassawi-1* is set to be the reference.

2 Intravarietal InDels are detected based on the raw sequencing reads.

We also compared the cp genome of *Hassawi-1* with those of *93–11* and *Nipponbare*. Between the *Hassawi-1* and *93–11* cp genomes, there are 40 deletions, 37 of which are 1-bp deletions and the longest is a 6-bp one in an IRA region. Most of them are interspecific deletions. The cumulative length attributable to InDels is 49 bp, which is consistent with the overall length difference between *Hassaw-1* and *93–11*. However, we did not find any insertions or SNPs between *Hassawi-1* and *93–11*. Comparing to the *Nipponbare* cp genome, we identified 71 deletions, 40 insertions, and 110 SNPs in *Hassawi-1*; the longest deletion is 69 bp (D-69, position 8,551) and the longest insertion is 32 bp (I-32, position 17,734). We also identified 13 co-segregated SNPs, which are all located in LSC region (**Table 3**). Half of these co-segregation SNPs are transversions between GC and CG. The co-segregated SNPs represent the best candidate molecular markers devoid of sequencing errors [12]. The co-segregating SNPs, S-2 (position 27,469 in *Hassawi-1*) is located in *rpoC2*, which is a key diagnostic variation between two switchgrass ecotypes [17]. The intersubspecific polymorphism rates between *Hassawi-1* and *Nipponbare* are 0.082% and 0.083% for SNPs and InDels, respectively.

**Table 3:** InDels and SNPs in cp genomes of *Hassawi-1* when compared to *93–11* and *Nipponbare.*

Reference	InDel/SNP	Position inreference	Position in*Hassawi-1*	Sequence	IntravarietalInDel
*93–11*	D-2	49224	49208	TT	N
	D-4	78379	78351	TTTT	N
	D-6	80552	80517	TCTTTT	N
*Nipponbare*	I-7	5014	5014	CCTTTAT	N
	D-69	8548	8551	GAATCCTATTTTTGTTCTTATACCCATGCAATAGA GAGCGAGTGGGAAAAGGGAGGTTACTTATTTTTT	N
	D-4	12671	12605	GAGG	N
	S-2	12819	12750	TA->CC	N
	D-2	14009	13940	CA	N
	D-6	17389	17317	ATAGAA	N
	S-2	17746	17669	GG->AA	Y
	I-32	17810	17734	TTAACAAATTCTTAGAGTATTTCTGGTAGAAT	Y
	S-2	27516	27469	CG->GC	N
	S-2	39771	39724	GC->CG	N
	S-2	41920	41871	GC->CG	N
	S-3	40687	40640	ACT->GAA	N
	I-2	43607	43553	GT	N
	D-5	46099	46038	TTATA	N
	D-2	46184	46117	TT	N
	D-6	46550	46479	AAGAAA	N
	D-2	49283	49208	TT	N
	I-2	50641	50566	GC	N
	I-15	53857	53782	CGAATTCCTATAGTA	N
	S-2	57028	56968	TT->AG	N
	S-2	57040	56979	CT->AA	N
	D-11	57053	56991	AAAAAAAAGTT	N
	I-5	57644	57572	TAAAG	N
	I-5	60861	60794	TTGTA	N
	S-2	64660	64594	CG->GC	N
	S-2	64688	64622	CG->GC	N
	D-2	65614	65547	TT	N
	I-2	68610	68541	AT	N
	S-2	70224	70156	GC->CG	N
	S-2	70307	70239	AG->GA	N
	D-6	75976	75909	TTAATT	N
	S-2	77793	77723	CG->AC	N
	D-5	78423	78351	TTTTT	N
	I-3	104518	104437	ACA	N
	I-6	110509	110431	ATAACT	N

Note: Only InDels larger than 1bp and co-segregating SNPs are shown.

### Mt Genome Variations

We compared the mt genome of *Hassawi-1* with *Hassawi-2* as well as between the representative *indica* and *japonica* varieties (**Table 4**). The cumulative length difference attributable to InDels is 74 bp, which is the total difference in length between *Hassawi-1* and its hybrid. There are 39 insertion and only 9 deletion events in addition to 26 base substitutions. The deletion rate for mt genome in *Hassawi-2* is nearly 4 times lower than that of the insertion. There is the same rate (0.003%) between transitions and transversions in mt genome of *Hassawi-2*. We found a large insertion (I-44, 44bp) within the intergenic region between *rrn18* and *rpl2* in *Hassawi-2*, which could be used as a useful marker distinguish the two *Hassawi* cultivars.

**Table 4:** Number and frequency of sequence variations in mt genomes when *Hassawi-1* is used as the reference and compared to *Hassawi-2*, *93–11* and *Nipponbare.*

	*Hassawi-2*	*93–11*	*Nipponbare*
SNP	26 (0.006%)	101 (0.022%)	233 (0.051%)
Transition	13 (0.003%)	57 (0.012%)	99 (0.022%)
Transversion	13 (0.03%)	44 (0.010%)	134 (0.029%)
InDel	48 (0.011%)	63 (0.014%)	150 (0.033%)
Insertion	39 (0.009%)	10 (0.002%)	61 (0.013%)
Deletion	9 (0.002%)	52 (0.012%)	89 (0.020%)
Total	74 (0.017%)	164 (0.036%)	383 (0.084%)

Unlike cp genomes, we identified 101 SNPs, 52 deletions, and 10 insertions in the mt genome between *Hassawi-1* and *93–11.* The presence and absence of deletions and SNPs within mt and cp genomes, respectively, confirm the different evolution characteristics between cp and mt genomes. As in *Hassawi-1* and *93–11*, deletion events in cp genomes occur at a rate of 0.03%, which is about 2.5 times higher than in mt genomes. The different evolution rate between cp and mt genomes in rice are also reported between *93–11* and a rice cultivar *PA64s* [24]. The fact that cp genome variation rate is higher than mt genome has also been confirmed based on a comparison between the *Hassawi* rice and *Nipponbare.* Between the mt genome of *Hassawi-1* and *Nipponbare*, the intersubspecific polymorphism rate for mt genomes is 0.051% for SNPs and 0.033% for InDels, nearly 1.5 and 2.5 times lower than that of their cp counterparts, respectively. There are a total of 383 intersubspecific polymorphisms (SNPs and InDels) identified between them, and we have not yet found any hotspots among the mt genomes. The transition and transversion rates are almost equal in mt genomes of different rice cultivars. Compared *Hasswi-1* with *93–11* and *Nipponbare*, there are a total of 14 large Indels (>10 bp) (**Table 5**). There are four common deletions exist in *Hassawi-1* (D-19, D-15, D-12, and D-13).

**Table 5:** Large InDels (>10bp) in mt genomes between *Hassawi-1* and *93–11* and between *Hassawi-1* and *Nipponbare.*

Reference	InDel	Position in reference	Position in *Hassawi-1*	Sequence
*93–11*	D-19	63127	195	CTGATTCAATAATAGAAGC
	D-15	235742	106227	AATGGAAAAAGAGTG
	D-12	235769	106237	CCCAAAAAAGGC
	D-13	235791	106244	GAGAAGGAGATAG
	D-16	243184	113610	CCGTCAGAGGCAGAAG
	D-21	289897	218077	ATAATTCGACAATTGCTGAGT
	I-10	360901	289056	ATTGGAGAAT
*Nipponbare*	D-19	62582	195	CTGATTCAATAATAGAAGC
	I-27	206165	115095	GCTTTGCCTGCTTCCTTAGCTACGTCA
	D-15	234791	106227	AATGGAAAAAGAGTG
	D-12	234818	106237	CCCAAAAAAGGC
	D-13	234840	106244	GAGAAGGAGATAG
	D-16	242238	113606	GAAGCCGTCAGAGGCA
	I-12	247610	404313	TAATATTCTTAT

### Microsatellites or Simple Sequence Repeats (SSRs)

SSRs, also known as microsatellites, have been used as genetic markers for evolutionary studies on organellar genomes due to their high variability [13], [25]. The complete SSR information on both cp and mt genomes of *Hassawi* rice and other varieties are summarized in **Table 6**. On average, the *Hassawi* rice cp genome has 4.3% SSR sequences with a density of 43.3 bp/kb. There are similar numbers of SSRs in the cp genome of the *indica* (*9311, Hassawi-1*, and *Hassawi-2*) and *japonica* (*Nipponbare*) varieties: 870 and 876 SSRs, respectively. Most SSRs are found in intergenic regions of the cp genome. Compared to *93–11*, the *Hassawi* rice cultivars have their SSRs more in genic and less in intergenic regions of the cp genome; the former one has SSR densities of 1.9/kb and 4.6/kb in genic and intergenic regions, respectively, and the latter two have the same corresponding SSR densities– 2.6/kb and 3.9/kb. As in the mt genome, similar results are observed. The SSR densities of *9311* are 0.5/kb and 4.6/kb in the genic and intergenic regions, respectively, and the corresponding numbers are 0.7/kb and 4.5/kb in the *Hassawi-1* mt genome. Compared to cp genomes, mt genomes possess both a lower percentage (3.6%) and a lower density (36 bp/kb) of SSRs in *Hassawi* rice and its hybrid over the *japonica* variety. Dinucleotide repeats are dominant in mt genomes whereas mononucleotide repeats are more frequently found in chloroplast genomes. Moreover, the mt genomes possess more tera-, penta-, and hexa-nucleotide repeats than the cp genomes. These findings are consistent with a previous observation [13].

**Table 6:** Distribution of SSRs in the four rice organellar genomes.

	Chloroplast				Mitochondrion			
**Repeat motif**	***93–11***	***Nipponbare***	***Haasawi-1***	***Hassswi-2***	***93–11***	***Nipponbare***	***Hasswi-1***	***Hassawi-2***
Mono-								
Genic	154	216	190	190	116	106	149	142
Intergenic	394	338	358	358	890	897	824	835
Di-								
Genic	79	135	126	126	118	96	140	134
Intergenic	190	135	143	143	1083	1104	976	981
Tri-								
Genic	19	32	29	29	21	19	25	25
Intergenic	23	10	14	14	248	253	216	216
Tetra-								
Genic	2	3	3	3	2	2	3	3
Intergenic	8	6	6	6	40	40	33	33
Penta-								
Genic	0	0	0	0	0	0	0	0
Intergenic	1	0	1	1	8	10	11	11
Hexa-								
Genic	0	0	0	0	1	1	1	1
Intergenic	0	1	0	0	0	0	0	0
Total	870	876	870	870	2527	2528	2378	2381
Frequency per kb	6.5	6.5	6.5	6.5	5.1	5.2	5.2	5.2
Density (bp/kb)	43.6	43.9	43.3	43.3	35.4	35.6	36.0	36.1

### Reverse Complementary Variations (RCVs) in Cp Genome

**Figure 5:**
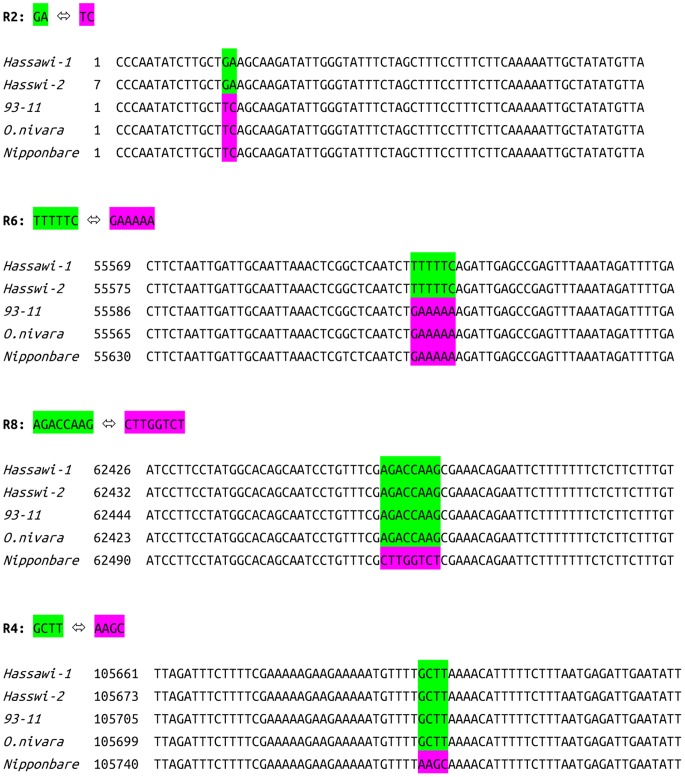
Detail alignments of reverse complementary variations in four cp genomes. The forward fragment is shown in green and the reverse fragment is shown in purple.

In addition to SNPs and InDels identified in cp genomes of different rice varieties and cultivars, we also noticed a novel type of sequence variations: reverse complementary variations (RCVs). RCV usually exists as a segment (>1 bp) in one sequence but its reverse complementary form is detected in the other. RCVs in rice have not been reported nor in any other plants. As shown in **Table 7**, there are two RCVs between *Hassawi-1* and *93–11* and four between *Hassawi-1* and *Nipponbare*. The detail alignments of those RCVs are presented in **Figure 5**. Only one of them is intravarietal in *Hassawi-1*. All of them are in LSC region except R-4 that is in SSC region (position 105,696 in *Hassawi-1*). Moreover, all RCVs are located in intergenic regions of cp genome except R-4 (position 105696 in *Hassawi-1*) in *ccsA*, which do not cause null mutations. The function of two genes (*accD* and *ccsA*) involving in RCVs is classified as miscellaneous. The RCV rate between *Hassawi-1* and *Nipponbare* is 0.003%, which is nearly 27 and 28 times lower than those of SNPs and InDels, respectively. The longest RCV in *Hassawi-1* is 8 bp (R-8, position 62,457) in intergenic region between *psbE* and *petL*. As a unique RCV in *Hassawi-1*, R-6 (position 55,604, TTTTTC), is a useful genetic marker to distinguish the two major rice subspecies. Since plant chloroplasts have their organelle-specific replication and DNA repair systems, the generation of RCVs may be related to these two systems. Since we did not identify any RCVs between *93–11* and a wild rice *Oryza nivara*, it is very suggestive that this RCV is either created as very rare event or the mechanism for its generation is developed later in the evolution of rice cp genomes.

**Table 7:** Reverse complementary variations (RCVs) in cp genomes when *Hassawi-1* is compared to *9311* and *Nipponbare.*

Reference	RCV^1^	Position in reference	Position in *Hassawi-1*	Sequence^2^	intravarietalRCV	Gene locus	Region
*93–11*	R-2	15	15	**TC->GA**	Y	*rps19-psbA*	LSC
	R-6	55621	55604	**GAAAAA->TTTTTC**	N	*rbcL-accD*	LSC
*Nipponbare*	R-2	15	15	**TC->GA**	Y	*rps19-psbA*	LSC
	R-6	55665	55604	**GAAAAA->TTTTTC**	N	*rbcL-accD*	LSC
	R-8	62521	62457	CTTGGTCT->AGACCAAG	N	*psbE-petL*	LSC
	R-4	105775	105696	AAGC->GCTT	N	*ccsA*	SSC

Note: 1. RCV stands for reverse complementary variation.

2. *Hassawi* specific RCVs are highlighted in bold.

### Repeats in Mt Genomes

Plant mitochondria have slow evolutionary rate and rapid rearrangement [26], [27]. Compared with plastid genomes, plant mt genomes are typically rich in large repeats. The extensive use of DNA recombination is an importance process in plant mt genome. Recently, DNA recombination in plant organellar genomes has been confirmed to play an important role in maintaining genome stability [28]. Moreover, there is ample evidence demonstrating that mt genome is important for plant sexual reproduction [29]. In rice, it has been proved that some novel mt genomic rearrangements are unique in cytoplasmic male sterility (CMS), where length variation of mt genome was observed [29]. Such DNA recombination has also been identified between wheat K-type cytoplasmic male sterility line and its maintainer line [26].

**Figure 6:**
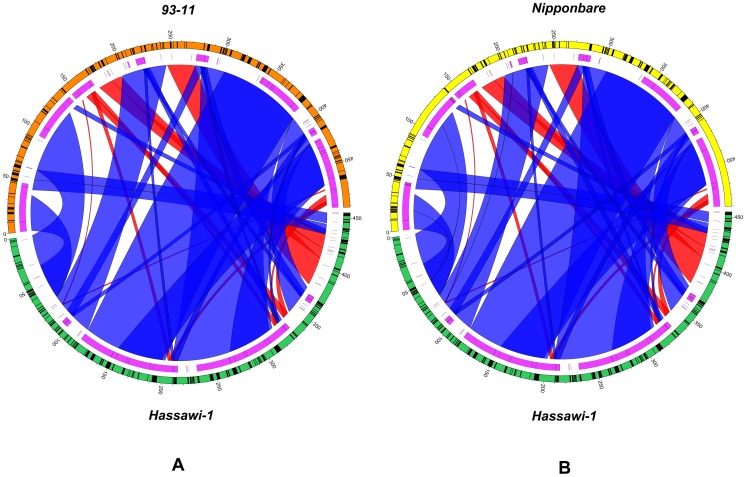
Circos diagram illustrating genome rearrangement and repeat distribution of mt genomes between *Hassawi-1* and *93–11* (A) and between *Hassawi-1* and *Nipponbare* (B). The first circle (from outside) displays different genomes (color-coding) and genes (blocks). The second circle displays repeat distribution along genomes. The connecting lines inside the circles join syntenic regions with direct (blue) and reversed matches (red) between two genomes.

Compared to other plants, rice mt genome has a higher content of repetitive sequences; there are 287,556 bp (58.5%) and 293,120 bp repeat sequences (59.7%) in *93–11* and *Nipponbare*, respectively. The eleven and thirteen large repeats (>1 kb) in *indica* and *japonica* are 277,828 bp and 272,688 bp in length, respectively (**Table 8**). However, the number of large repeats is reduced to six in the mt genomes of *Hassawi-1* and its hybrid. Moreover, the lengths of these large repeats had been increased with the longest repeat of 96,168 bp and 96,165 bp in *Hassawi-1* and *Hassawi-2*, respectively. All large repeats in the *Hassawi* rice match in the forward direction. Compared to *93–11* and *Nipponbare*, the structure of the *Hassawi* mt genomes had been re-organized to accumulate more repeats in the large repeat regions (**Figure 6A**). This unusual genomic organization is also detectable by plotting the syntenic regions between *Hassawi-1* and either *93–11* or *Nipponbare* (**Figure 6B**). Longest repeats are clearly identifiable, which are accounted for 78% of the total repeat length in both *Hassawi-1* and *Hassawi-2*. The counts for functional genes in mt genomes are highly conserved among the *Hassawi* rice and other varieties, but the function of the lost sequences remains unknown. The dynamic genomic rearrangement may represent responses to environment pressures during mt genome evolution of the *Hassawi* rice and results in an enrichment of large repeats and deletions of some functionally unknown repetitive sequences.

**Table 8:** Large repeats (>1 kb) in mt genomes of the four rice cultivars.

Cultivars	Length(bp)	Startposition 1	Direction^1^	Startposition 2
*Hassawi-1*	96165	115395	F	233158
	6123	208572	F	363729
	6030	91768	F	326335
	4078	90678	F	362639
	2988	91768	F	208572
	2988	326335	F	363729
*Hassawi-2*	96168	115369	F	233177
	6124	208548	F	363754
	6031	91738	F	326356
	4079	90648	F	362664
	2989	91738	F	208548
	2989	326356	F	363754
*93–11*	46082	341366	F	422479
	37257	0	F	95060
	15084	153411	F	476431
	9917	37259	F	132319
	8800	215462	F	274582
	7214	279303	F	410259
	6322	145536	F	468556
	4079	220183	F	410259
	1551	151859	F	474879
	1304	149275	P	207170
	1304	207170	P	472295
*Nipponbare*	45584	340412	F	421499
	43760	0	F	94527
	10305	157114	F	480215
	10028	147085	F	470186
	5865	279707	F	410628
	4078	219233	F	409280
	3660	219651	F	409698
	3103	214512	F	273638
	2730	220581	F	279707
	2625	144459	F	467560
	2034	217616	F	276742
	1347	278359	F	409280
	1225	45050	F	139577

Note: 1. F and P stand for forward and palindromic matches, respectively.

### The Origin of the Hassawi Rice

**Figure 7:**
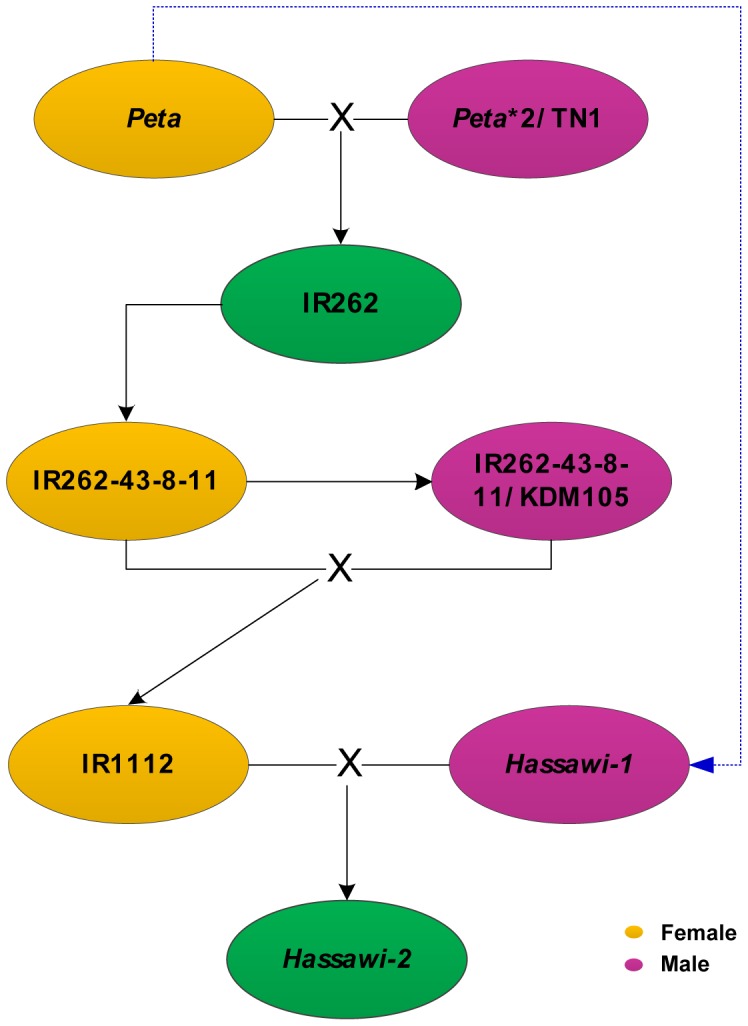
The origins of *Hassawi* rice and it’s hybrid. The blue line predicts the origin of wild-type *Hassawi* rice from the Indonesian variety *Peta*.

**Figure 8:**
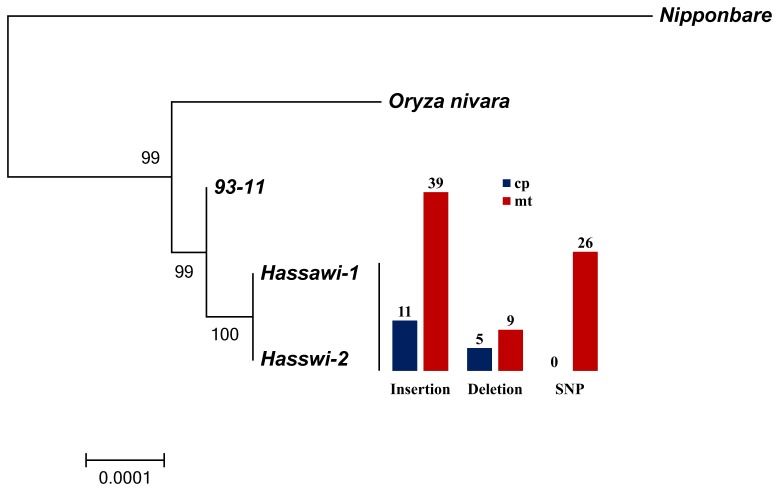
Phylogenetic relationships of 5 rice cultivars as determined from whole cp genomes. Support values are shown for nodes as maximum likelihood bootstrap. The scale bar denotes substitutions per site. The histogram presents the variations (Insertion, deletion and SNP) in both cp and mt genomes between *Hassawi-1* and *Hassawi-2*.

According to the breeding record (**Figure 7**), *Hassawi-2* is a hybrid between the wild-type *Hassawi-1* and an *indica* cultivar IR1112 (the International Rice Research Institute, IRRI). There has been a limited literature about the genetic background of both *Hassawi* rice cultivars. Tracing back to IR1112, we found that IR1112 is a cross between IR262-43-8-11(maternal parent) and IR262-43-8-11/KDM105 (paternal parent), and both of its parental cultivars are descendants of IR262. From a cross of *Peta* and Peta*2/TN1, IR262 has a maternal inheritance of *Peta* that is an *indica* variety from Indonesia. The molecular phylogeny analysis based on whole cp genomes of five rice cultivars including wide rice *Oryza. nivara*, showed that both *Hasswi-1* and *Hassawi-2* had a common *indica* ancestor, which was closely related to *O. nivara* (**Figure 8**). Moreover, recent research about resequencing 50 accessions of cultivated and wild rice revealed that *indica* was very closely related to *O.nivara*, whereas *japonica* was closer to *Oryza. rufipogon* and father from *O.nivara* [30]. The chloroplasts, together with mitochondria of higher plants, are maternally inherited and have their specific replication and DNA repair systems [24], [31], whereas the nuclear genome is bi-parentally inherited. With uniparental inheritance, organellar genomes are often used for tracing phylogenetic relations [31]. Examining variations in both cp and mt genomes between *Hassawi-1* and *Hassawi-2* (**Figure 8**), we conclude that *Hassawi-1* and IR1112 are the paternal and maternal parents of *Hassawi-2*, respectively, and the cp and mt genomes of *Hassawi-2* are inherited from *Peta* in distant origin. Considering the divergence of rice organellar genomes among all the analyzed *indica* or *japonica* varieties and between *Hassawi-1* and *Hasswi-2*, we suggest that the wild-type *Hassawi* rice is a descendant of *Peta*, which had adapted to the current or similar environment some hundreds or even thousands of years ago and it would be interesting to know the particular history and origin of the *Hassawi* rice for the sake of both science and civilization studies. Nevertheless, understanding the inheritance of the wild-type *Hassawi* and its hybrid provides important genetic information for their future breeding as well as genetic and molecular studies.

### Conclusion

We report here the complete cp and mt genome assemblies of the wild-type *Hassawi* and its hybrid, and demonstrate their high degree of conservation in gene content and order among the sequenced rice varieties. Although functional genes among rice mt genomes are also conserved, their gene order, genome structure, and genome size are often variable. Our analyses on sequence variations, including SNPs, InDels, and RCVs in both cp and mt genome assemblies of the *Hassawi* rice provide detailed genetic information for genetically differentiating the two *Hassawi* cultivars. A greater number of large and enriched repeats are found in the *Hassawi* mt genomes as compared to other sequenced rice varieties. This observation is also supported by the distribution of SSRs, which also shows a higher density in the *Hassawi* rice when compared to those of other rice varieties. Recombination of mt genomes is prevalent in the *Hassawi* rice and results in complex genome rearrangements. As in the other plant, this phenomenon leads us to believe that such a repeat redistribution in mitochondrial genome may play a role in maintaining genome stability. As a final note, sequence variation data acquired in this study provide strong evidence for the origin of the *Hassawi* rice and its hybrid: both may be descendents of an Indonesian variety *Peta* albeit through different routes and at different time in history.

## Materials and Methods

### Genome Sequencing and Assembly

Both *Hassawi* rice cultivars were collected from Al-Hassa, Kingdom of Saudi Arabia. We extracted genomic DNA from 50 g young green leaves according to a CTAB-based method [32] and constructed libraries according to the GS FLX Titanium general preparation protocol, started with 5 g purified DNA. The ssDNA libraries were amplified with emulsion-PCR and enriched, and the samples were sequenced on Roche/454 GS FLX platform. In addition, two mate pair libraries for both cultivars were constructed by following SOLiD Library Preparation Guide (SOLiD 4.0). 20 µg or more genomic DNA was used for sequencing in SOLiD 4.0 instrument, which depending on two different insert sizes (500–1000 bp and 1000–3000 bp).

We extracted cp and mt genome sequence reads from whole genome sequencing data generated from both 454 GS FLX and SOLiD 4.0 platforms and assembled the 454 GS FLX reads based on a protocol we developed recently[14]. For the cp genome assembly, we filtered cp reads from the raw data according to the three known rice cp genome sequences. The clean cp reads were assembled into contigs by using Newbler (v2.6). In the mt genome assembly, we first assembled the raw data with Newbler, and then used Blast tool to filter for the mt contigs that were aligned to the known rice mt genomes. The mt contigs are not usually clean enough as they often contain cp genome sequences. We also used information on unknown mt contigs and read coverage for the removal of cp sequence contaminations. At the end, we validated the organellar genome assemblies with SOLiD sequencing data.

### Genome Annotation

We used DOGMA for cp genome annotation (Dual Organellar GenoMe annotator)[33] and manually corrected start and stop codons. We annotated mt genome based on aligning sequences to the known rice mt genomes using NCBI BlastX and BlastN tools. We carried out all BlastN and BlastX searches using the blastall executable (version 2.2.25) with default settings (e-value 1e-10). Protein-coding genes, rRNAs, and tRNAs were identified by using the plastid/bacterial genetic code. We also used tRNAscan-SE[34] to corroborate tRNA boundaries identified by BlastN.

### Genome Comparison Analysis

SSRs were identified and localized by using the Simple Sequence Repeat Identification Tool (SSRIT) [35] that identifies perfect nucleotide repeats of mono-, di-, tri- tetra-, tetra-, penta-, and hexa-nucleotides, and those equal or greater than three repeat units were collected except monomers. Intersubspecific polymorphisms were first identified based on the MUMmer package (v3.06) [36]. The results were then acquired by using a custom-designed Perl script and confirmed through careful visual inspection. Intravarietal polymorphisms were identified by using Newbler (v2.6) and Bioscope (v1.3) software, for 454 data and SOLiD data, respectively. We carried out repeat sequence analysis using the REPuter web-based interface (http://bibiserv.techfak.uni-bielefeld.de/reputer) [37], including forward, palindromic, reverse, and complemented repeats with a minimal length of 50 bp. Cp-derived sequences are identified with BlastN search of mt genomes against annotated cp genomes (Identity ≥80%, E-value ≤1e-5, and Length ≥50 bp). The cp-derived sequences were then aligned to all known plant mt genomes by using BlastN (Identity ≥80%, E-value ≤1e-5, and Coverage ≥50%). The syntenic regions of cp and mt genomes between different cultivars were detected by using Nucmer of the MUMmer package (v3.06) [36] with 50-bp exact minimal match. The annotated cp and mt genome features including gene coordinate, genome structures in cp genomes, repeats in mt genomes and different genome variations were used to draw genome maps using Circos software [38].

### Phylogenomic Analysis

The whole cp genomes of five rice cultivars were aligned using the program MAFFT version 6 [39] and adjusted manually where necessary. The unambiguously aligned DNA sequences were used for phylogenetic tree construction. Maximum likelihood method analysis was performed with PhyML v3.05 [40] under GTR (General time Reversible) model of nucleotide substitution to construct phylogenetic tree. 1,000 bootstrap replications were used to estimate the confidence of brand points. We obtained the best tree after heuristic search with the help of Modelgenerator [41].

### Accession Numbers

The complete sequence of the four genomes was deposited to GenBank with accession numbers: JN861109, JN8611010, JN8611011, and JN8611012 for *Hassawi-1* chloroplast, *Hassawi-2* chloroplast, *Hassawi-1* mitochondrial, and *Hassawi-2* mitochondrial genomes, respectively. Other sequences used for comparative analysis are: NC_008155, NC_001320, NC_005973, NC_007886, and NC_011033 from *93–11* chloroplast, *Nipponbare* chloroplast, *O. nivara* chloroplast, *93–11* mitochondrial, and *Nipponbare* mitochondrial genomes, respectively.

## Supporting Information

Figure S1 **The cp genome assembly of** ***Hassawi-1*** **from 454 sequencing reads.** The large single copy, the small single copy, and the inverted repeats are shown in blue, green, and red, respectively. The boxes stand for contigs and the lines indicate the link (overlapping) between two contigs. The numbers in the boxes show contig name, length, and read depth. The numbers on lines are reads spanning two contigs. This figure was generated with Graphviz (http://www.graphviz.org/).
(PDF)

Figure S2 **The cp genome assembly of** ***Hassawi-2*** **from 454 sequencing reads.** The large single copy, the small single copy, and the inverted repeats are shown in blue, green, and red, respectively. The boxes stand for contigs and the lines indicate the link (overlapping) between two contigs. The numbers in the boxes show contig name, length, and read depth. The numbers on lines are reads spanning two contigs.
(PDF)

Figure S3 **The mt genome assembly of** ***Hassawi-1*** **from 454 sequencing reads.** The boxes stand for contigs and the lines indicate the link (overlapping) between two contigs. The numbers in the boxes show contig name, length, and read depth. The numbers on lines are reads spanning two contigs. The boxes in red show contigs with matched the mt genomes of other rice cultivars.
(PDF)

Figure S4 **The mt genome assembly of** ***Hassawi-2*** **from 454 sequencing reads.** The boxes stand for contigs and the lines indicate the link (overlapping) between two contigs. The numbers in the boxes show contig name, length, and read depth. The numbers on lines are reads spanning two contigs. The boxes in red show contigs with matched the mt genomes of other rice cultivars.
(PDF)
