# Supplementary material for: The Organelle Genomes of Hassawi Rice (Oryza sativa L.) and Its Hybrid in Saudi Arabia: Genome Variation, Rearrangement, and Origins
Source: PLoS One. 2012 Jul 31;7(7):e42041. doi: 10.1371/journal.pone.0042041 (PMC3409126; doi:10.1371/journal.pone.0042041)

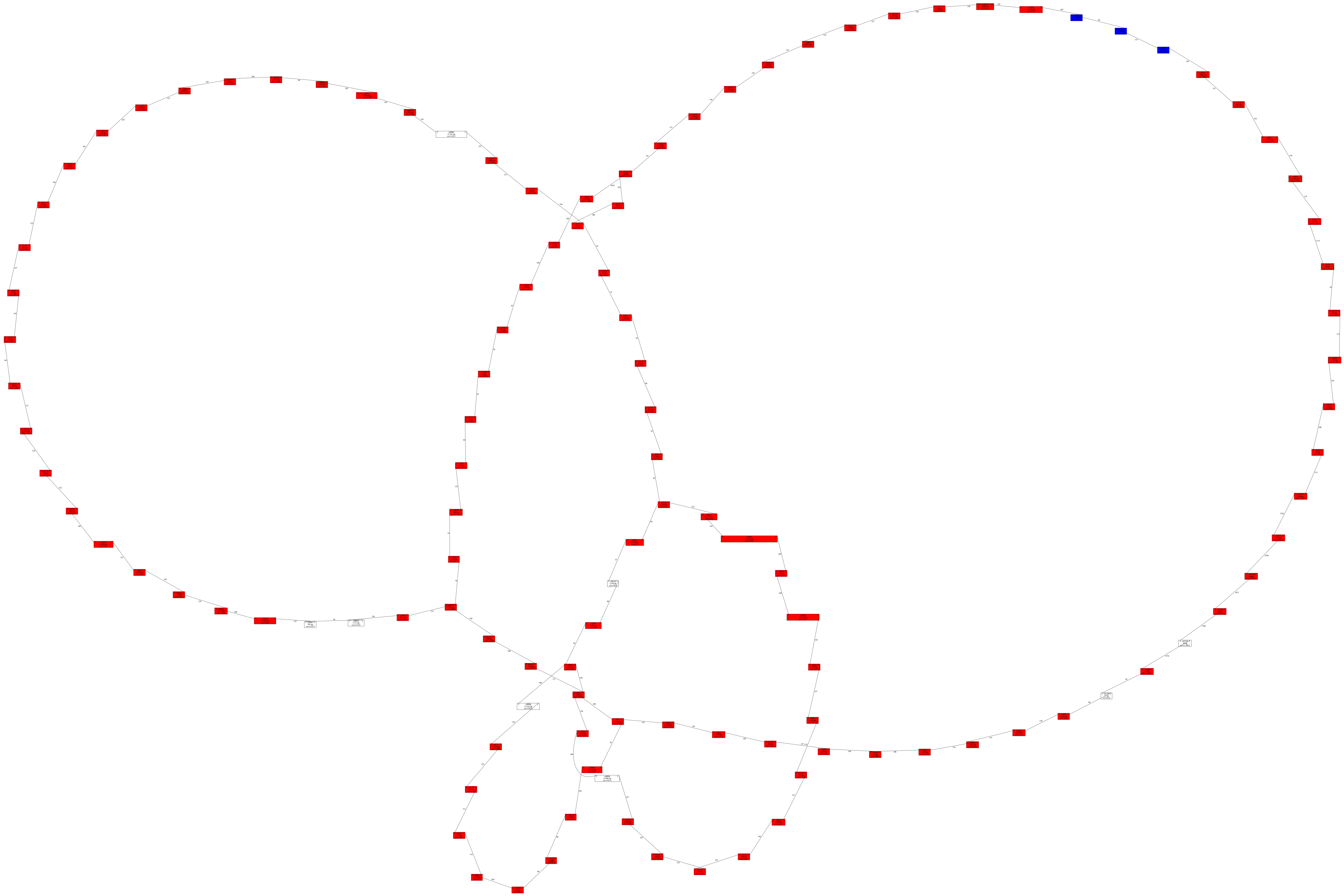

Supplement: Figure S3 — The mt genome assembly of Hassawi-1 from 454 sequencing reads. The boxes stand for contigs and the lines indicate the link (overlapping) between two contigs. The numbers in the boxes show contig name, length, and read depth. The numbers on lines are reads spanning two contigs. The boxes in red show contigs with matched the mt genomes of other rice cultivars. (PDF) [file pone.0042041.s003.pdf]
